# Kind Words

**Published:** 1886-12-20

**Authors:** 


KIND WORDS.—We get many compliments from the appreciative readers of our journal, which serve to encourage us greatly. In a letter received from Dr. E. P. Overby, of Georgia, he says of the RECORD: " One of the best journals

I ever saw." And away over in Oregon, Dr. W. A. Cusic writes: "The RECORD is one of the limited number of medical journals which is uniformly and always good." Dr. W. N. Mahan writes: "The RECORD comes nearer filling the every-day wants of the physician than any journal I have ever read," etc.

---

LIBERAL OFFER.—Every subscriber who, on renewing his subscription for 1887, will send us a new subscriber will be allowed club rates; or, for $3 we will send the RECORD one year to himself and his friend. We hope our readers will bear this proposition in mind. If everyone will use a little effort in this way he can reduce his own subscription, and, at the same time, do us and himself a service by enlarging our list and enabling us to improve the RECORD, in which he, as a reader, is interested. Friends, don't forget this.

---

IMPERIAL GRANUM.—We indorse the following remark by one of our exchanges: *A Food that is Medicine.*—Some years ago our attention was called to the value of Imperial Granum as a food particularly serviceable to children and to all who suffer from disorders incident to mal-assimilation. We were then able to praise it very warmly, and now, after many years, we are able, with increased confidence, to renew our commendation of it to our readers. The principle on which it is prepared is good, and the effects from its use, we have observed, have been excellent.

---

LUNATIC ASYLUM.—We are in receipt of a copy of the Report of the Trustees and other officers of the Lunatic Asylum of the State of Georgia, at Milledgeville, from October 1, 1885, to October 1, 1886.

The following are its officers: Superintendent and Resident Physician, Dr. T. O. Powell; First Assistant Physician, Dr. J. M. Whitaker; Second Assistant Physician, Dr. Harris Hall; Third Assistant Physician, Dr. L. M. Jones; Fourth Assistant Physician, Dr. M. H. O'Daniel; Apothecary, P. A. West; Steward, L. J. Lamar; Assistant Steward, G. W. Hollingshead; Secretary, Fleming G. Grieve; Matron, Mrs. J. M. Darnell; Treasurer, Thos. T. Windsor; Chaplain, Rev. A. J. Beck.

From the report of Dr. T. O. Powell, the faithful and efficient superintendent, we extract the following interesting items:

There were present, October 1, 1886, white male lunatics, 353; white male epileptics, 48; white male idiots, 15—total white males, 416. White female lunatics, 412; white female epileptics, 40; white female idiots, 20—total white females, 472—total whites, 888.

